# TSPO Modulation by PIGA-1138 Attenuates Oxidative Stress and Preserves Retinal Function in Experimental Diabetic Retinopathy

**DOI:** 10.3390/antiox15081000

**Published:** 2026-08-12

**Authors:** Alessia Galante, Francesca Corsi, Rosario Amato, Sabrina Taliani, Federico Da Settimo, Maurizio Cammalleri, Ilaria Piano, Massimo Dal Monte, Claudia Gargini

**Affiliations:** 1 Department of Pharmacy, University of Pisa, 56126 Pisa, Italy; alessia.galante@phd.unipi.it (A.G.); francesca.corsi@farm.unipi.it (F.C.); sabrina.taliani@unipi.it (S.T.); federico.dasettimo@unipi.it (F.D.S.); maria.gargini@unipi.it (C.G.); 2 Department of Biology, University of Pisa, 56126 Pisa, Italy; rosario.amato@unipi.it (R.A.); maurizio.cammalleri@unipi.it (M.C.); 3 Interdepartmental Center Pisa Neuroscience “PiNeuro”, University of Pisa, 56126 Pisa, Italy

**Keywords:** PIGA-1138, neuroprotective ligands, hyperglycemic insult, visual electrophysiology, metabolic neurodegeneration

## Abstract

**Introduction:** Diabetic retinopathy (DR) is characterized by early retinal neurodegeneration accompanied by progressive alterations of the retinal microvasculature, both exacerbated by hyperglycemia-induced oxidative stress and inflammation. Mitochondrial dysfunction critically contributes to neuronal loss and vascular impairment. The 18 kDa Translocator Protein (TSPO) is a mitochondrial outer membrane protein whose expression is increased in activated retinal glial cells and represents a promising target to modulate neuroinflammation and oxidative stress. This study evaluates the therapeutic potential of the TSPO ligand PIGA-1138 in experimental models of DR. **Methods:** PIGA-1138 (3 µM in vitro; 10 mg/kg/day, i.p., in vivo) was evaluated in high glucose (HG)-exposed 661W retinal cells and in streptozotocin (STZ, 150 mg/kg)-induced diabetic C57BL/6J mice. Cell viability, mitochondrial function, oxidative stress, and Nrf2, HO-1, and SOD1 expression were assessed in vitro. Retinal function and morphology were evaluated in vivo by electroretinography (ERG), visual acuity testing, and optical coherence tomography (OCT) at 30 and 60 days after diabetes induction. **Results:** PIGA-1138 significantly improved cell viability, reducing apoptosis (TUNEL *p* ≤ 0.01), preserving mitochondrial membrane potential (MitoRed *p* ≤ 0.01), reducing oxidative damage, and enhancing Nrf2 nuclear translocation together with HO-1 (*p* ≤ 0.05) and SOD1 (*p* ≤ 0.01) expression in HG-treated retinal cells. In diabetic mice, treatment preserved ERG responses and limited retinal thinning at 60 days (*p* ≤ 0.01), while showing a trend toward preserving visual acuity. **Conclusions:** Targeting mitochondrial TSPO with PIGA-1138 attenuates key hallmarks of DR by mitigating oxidative stress, suppressing neuroinflammation, and preserving retinal structure and function. These findings support TSPO as a potential disease-modifying target for DR.

## 1. Introduction

Neurodegeneration and microvascular dysfunction represent the two major and interdependent pathological hallmarks of Diabetic Retinopathy (DR), one of the leading causes of vision loss worldwide, and is currently recognized as a multifactorial neurovascular disease. Rather than being exclusively a microvascular complication of diabetes, DR involves the progressive and interconnected impairment of retinal neurons, glial cells, and the microvasculature, all of which are adversely affected by chronic hyperglycemia [1,2,3]. Sustained high-glucose (HG) conditions promote excessive production of reactive oxygen species (ROS) through mitochondrial dysfunction, NADPH oxidase activation, and impaired antioxidant defenses, leading to oxidative stress that critically contributes to both neuronal loss and blood–retina barrier breakdown [4,5,6].

Oxidative stress and chronic inflammation are inextricably linked in the pathogenesis of DR. Hyperglycemia-induced excessive generation of ROS triggers redox-sensitive signaling pathways that activate a molecular network, finally leading to the increased expression of pro-inflammatory cytokines that compromise the integrity of retinal tissue [7,8]. In this scenario, the innate immune response—partially mediated by microglia—undergoes a transition toward a chronic, maladaptive phenotype. This activation contributes to the amplification of oxidative stress and the release of neurotoxic mediators, fueling a vicious cycle that culminates in neuronal apoptosis and the vascular instability characteristic of the disease [9,10].

In this context, mitochondria emerge as central hubs linking metabolic imbalance, neurodegeneration, and vascular damage. A key element of the outer mitochondrial membrane is the 18 kDa Translocator Protein (TSPO), involved in cellular homeostasis, cholesterol transport, and neurosteroid biosynthesis. Although expressed at low levels in the healthy retina, TSPO is markedly upregulated across various retinal cell populations (including microglial and immune cells) in response to degenerative stimuli, making it a reliable biomarker of inflammatory status and a promising pharmacological target [9,11]. In addition, although the presence of TSPO in retinal neurons has been questioned [12,13], its expression has been demonstrated in a murine photoreceptor-like cell line [14].

Pharmacological modulation of TSPO through selective ligands exerts antioxidant and neuroprotective effects that include the activation of the transcription factor nuclear factor erythroid 2-related factor 2 (Nrf2) and its target genes such as superoxide dismutase 1 (SOD1) and heme oxygenase 1(HO-1), which, all in all, act as an important antioxidant defense system [15,16]. TSPO modulation also stimulates mitochondrial neurosteroidogenesis. The production of pregnenolone and its derivatives bolsters endogenous defenses against apoptosis and oxidative stress [17,18]. By acting on mitochondrial stability, TSPO modulation limits ROS production and mitigates cytokine release, ultimately preserving neuronal viability and vascular functionality through systemic regulation of the retinal microenvironment [19,20].

PIGA-1138 is a TSPO ligand rationally designed to exhibit high binding affinity, prolonged residence time at the TSPO binding site, and enhanced neurosteroidogenic efficacy. Preclinical studies have demonstrated that PIGA-1138 effectively increases endogenous neurosteroid levels and exerts potent anti-inflammatory and neuroprotective actions in models of autoimmune encephalomyelitis, neuroinflammation, and cellular stress [21]. More recently, PIGA-1138 has been shown to attenuate oxidative stress and prevent neurodegeneration in in vitro and in vivo models characterized by mitochondrial dysfunction and chronic inflammation, and its TSPO-mediated neuroprotective activity has been validated in retinal cellular models through comparison with the reference TSPO ligand PK11195 [14,22].

Given the central role of oxidative stress, microglial activation, and mitochondrial dysfunction in DR, targeting TSPO with PIGA-1138 represents a compelling therapeutic strategy to promote resilience in the diabetic retina. By attenuating microglial-driven inflammation, reducing ROS production, and sustaining endogenous neurosteroid synthesis, TSPO modulation may concurrently protect retinal neurons and preserve microvascular integrity—addressing both major pathological components of DR. However, despite this strong mechanistic rationale, direct evidence supporting the efficacy of TSPO ligands in diabetic retinal models remains limited. Therefore, here we test the efficacy of TSPO targeting PIGA-1138 for the treatment of DR, providing evidence of a putative intracellular signaling pathway involved in neural cell protection using in vitro culture of retinal neuronal cells exposed to HG conditions. We further extended the efficacy analysis to an in vivo murine model of DR, testing the beneficial efficacy of PIGA-1138 on retinal function through electrophysiological recordings and behavioral tests, as well as on retinal structure through the in vivo measurements of retinal layer thickness.

## 2. Materials and Methods

### 2.1. Cell Culture

The 661W photoreceptor-like cells were obtained from Dr. Muayyad Al-Ubaidi (University of Oklahoma Health Sciences Center). Cells were cultured in HG Dulbecco’s Modified Eagle’s Medium (DMEM HG) supplemented with 10% fetal bovine serum and 1% penicillin/streptomycin. Cultures were maintained at 37 °C in a humidified atmosphere containing 5% CO_2_. All reagents and materials used for cell culture maintenance were purchased from Merck KGaA (Darmstadt, Germany), unless otherwise specified.

A batch of PIGA1138 was freshly prepared essentially following the synthetic procedure previously described [23]. PIGA-1138 was initially dissolved in dimethyl sulfoxide (DMSO) to obtain a 25 mM stock solution, which was subsequently diluted in high-glucose DMEM to a final concentration of 3 µM.

A 1 M glucose stock solution was prepared by dissolving D-(+)-glucose in Milli-Q water and then sterile-filtering. The glucose solution was added to DMEM HG at a final concentration of 30 mM, resulting in a total glucose concentration of 55 mM, considering the basal 25 mM glucose already present in DMEM HG [24].

A 1 M sucrose stock solution was prepared by dissolving sucrose in Milli-Q water and then sterile-filtering. The sucrose solution was added to DMEM HG at a final concentration of 30 mM. This solution was used to exclude osmotic effects; cells in the control (CTRL) group were supplemented with sucrose to match the osmolarity of the HG medium (Figure S1).

### 2.2. Cell Treatments

661W cells were seeded in 96-well multiwell plates or chamber slides at a density of 1 × 10^4^ cells/well and allowed to adhere for 24 h. Cells were then exposed to HG, 55 mM, for 24 h to induce cellular damage. After this initial damage phase, cells were treated for an additional 24 h with PIGA-1138 (3 μM) in the continued presence of HG. CTRL cells were maintained under normoglycemic conditions throughout the experiment. Cells were additionally treated with SU-10603 (3 μM), an inhibitor of pregnenolone metabolism, administered together with PIGA-1138 during the second 24 h treatment period, in the continued presence of HG. SU10603 was used as a pharmacological tool to investigate the contribution of TSPO-mediated neurosteroidogenesis to the protective effects of PIGA-1138.

### 2.3. Cell Viability Assay

Cell viability was determined using the CellTiter 96^®^ Aqueous One Solution Cell Proliferation Assay (cat #G3582, Promega, Madison, WI, USA), following manufacturer’s instructions. Cells were seeded in 96-multiwell plates and treated as described above. At the end of the treatments, the reagent was added directly to the culture medium and incubated at 37 °C for 2 h. Absorbance was measured using an EnSight™ Multimode Plate Reader (PerkinElmer, Waltham, MA, USA), and cell viability was expressed as a percentage relative to CTRL.

### 2.4. TUNEL Assay

Cells were seeded into 8-well chamber slides and treated as described above. At the end of the treatments, cells were fixed with 4% paraformaldehyde (PFA) for 25 min at room temperature and permeabilized with 0.2% Triton^®^ X-100 in phosphate-buffered saline (PBS) for 5 min. Nuclear DNA fragmentation was assessed using the DeadEnd™ Fluorometric TUNEL System (cat #G3250, Promega, Madison, WI, USA), following the manufacturer’s instructions. Nuclei were counterstained with 4,6-diamidine-2-phenylindole (DAPI; cat #D9542, Merck KGaA) at a 1:5000 dilution in PBS. Images were acquired using a Nikon Ni-E fluorescence microscope (Nikon Instruments Inc., Melville, NY, USA) equipped with a DS-Ri2 camera and analyzed using ImageJ 8.0 software (NIH, Bethesda, MD, USA). TUNEL-positive cells were quantified by manually counting all cells within each field. The number of TUNEL-positive nuclei was then expressed as a percentage of the total number of DAPI-positive nuclei in the corresponding field.

### 2.5. Assessment of Mitochondrial Membrane Potential (MitoRed)

Changes in mitochondrial membrane potential (ΔΨm) were assessed using the fluorescent dye MitoRed (cat #53271 Merck KGaA). Cells were seeded and exposed to HG and PIGA-1138 as described above.

At the end of the treatments, cells were incubated with MitoRed at a final concentration of 50 nM at 37 °C for 60 min. After incubation, cells were gently washed with culture medium, and fluorescence was measured directly in the 96-well plate using an EnSight™ Multimode Plate Reader (PerkinElmer). Data were expressed as a percentage of fluorescence intensity relative to CTRL.

### 2.6. Assessment of Mitochondrial Membrane Potential (MitoLight)

The ΔΨm was additionally evaluated using the fluorescent probe MitoLight (cat #APT142 Merck KGaA). 661W cells were seeded into 8-well chamber slides and treated according to the cell treatment protocol described above.

At the end of the treatments, cells were incubated with MitoLight at the concentration recommended by the manufacturer at 37 °C for 15 min. Following incubation, cells were rinsed with culture medium and fixed in 4% paraformaldehyde (PFA) for 25 min at room temperature. Nuclei were subsequently counterstained with DAPI (Merck KGaA) diluted 1:5000 in PBS. Fluorescence images were captured using a Nikon Eclipse Ti A1-A confocal microscope (Nikon Instruments Inc.).

### 2.7. Western Blot Analysis

For protein expression analysis, 661W cells were seeded in a 6 cm Petri dish at a density of 3 × 10^5^ cells and treated according to the experimental protocol described above. At the end of the treatments, cells were washed with PBS and lysed in radioimmunoprecipitation buffer (150 mM NaCl, 50 mM Tris–HCl pH 8, 1% Igepal, 0.5% Na-deoxycholate, 0.1% SDS; and protease inhibitors—1 μM Orthovanadate and 0.1 mg/mL PMSF). Cell lysates were collected and clarified by centrifugation at 12,000× *g* for 30 min at 4 °C.

Protein concentration was determined using the DC™ Protein Assay (Bio-Rad Laboratories, Hercules, CA, USA), according to the manufacturer’s instructions.

Equal amounts of protein (30 μg) were mixed with 4× Laemmli buffer, separated on 4–20% precast polyacrylamide gels with stain-free technology (Mini-PROTEAN TGX, Bio-Rad Laboratories), and transferred onto PVDF membranes using the Trans-Blot Turbo system (Bio-Rad Laboratories, Hercules, CA, USA). Membranes were blocked with EveryBlot Blocking Buffer (Bio-Rad Laboratories, Hercules, CA, USA) for 15 min at room temperature and incubated overnight at 4 °C with a rabbit primary antibody anti-SOD1 (cat #SAB5200083, Merck KGaA). After washing with Tris-buffered saline containing 0.05% Tween-20 (TBS-T), membranes were incubated for 2 h at room temperature with a horseradish peroxidase-conjugated goat anti-rabbit secondary antibody (Bio-Rad Laboratories, Cat. No. 1706515). After incubation, immunoreactive bands were detected using Clarity™ Western ECL Substrate (Bio-Rad Laboratories) and acquired with a ChemiDoc XRS+ system (Bio-Rad Laboratories). Densitometric analysis was performed with Image Lab software version 6.0 (Bio-Rad Laboratories), and the intensity of each protein band was normalized to the total protein content.

### 2.8. RT-qPCR Analysis

For gene expression analysis, 661W cells were seeded in 6 wells at a density of 3 × 10^5^ cells and treated according to the experimental protocol described above. Total RNA purification and extraction were performed using the miRNeasy Micro Kit (Qiagen, Hilden, Germany) according to the manufacturer’s instructions. RNA concentration and purity were assessed using a NanoDrop Lite spectrophotometer (Thermo Fisher Scientific, Waltham, MA, USA). A total of 1 μg of RNA was used for retro-transcription reaction by using the iScript cDNA Synthesis Kit (Bio-Rad Laboratories).

For quantitative Real-Time PCR, 50 ng of cDNA per reaction was used following the manufacturer’s instructions. PrimePCR™ SYBR^®^ Green Assays for mouse *Hmox1* (Assay ID: qMmuCID0040051), *Actb* (Assay ID: qMmuCED0027505), and *Gapdh* (Assay ID: qMmuCED0027497) (Bio-Rad Laboratories) were used, following the manufacturer’s protocol. *Hmox1*, *Actb*, and *Gapdh* are the genes encoding HO1, β-actin and glyceraldehyde phosphate dehydrogenase, respectively. Gene expression levels were normalized to the average of *Actb* and *Gapdh* as housekeeping genes. Gene expression levels were calculated as fold change using the 2^−ΔΔCt^ method, relative to CTRL.

### 2.9. Immunofluorescence

NRF2 intracellular localization was evaluated by immunofluorescence analysis. 661W cells were seeded into 8-well chamber slides at a density of 1 × 10^4^ cells/well and treated according to the cell treatment protocol described above.

At the end of the treatments, cells were washed with PBS and fixed with 4% PFA for 25 min at room temperature. Cells were then permeabilized with 0.3% Triton X-100 in PBS for 10 min and blocked with 2.5% bovine serum albumin (BSA) in PBS for 1 h at room temperature to reduce nonspecific binding.

Cells were incubated overnight at 4 °C with rabbit primary anti-NRF2 (Cat #PA5-27882 Thermo Fisher Scientific, 1:500) or mouse anti-Acrolein (cat #ab48501 Abcam, Cambridge, UK) antibodies diluted in 1% BSA solution. After three washes in PBS, cells were incubated for 2 h at room temperature with the Alexa Fluor 568-conjugated secondary antibodies (cat #A-11011; #A-11004 Thermo Fisher Scientific). Nuclei were counterstained with DAPI (Merck KGaA) at a 1:5000 dilution in PBS. Slides were mounted using antifade mounting medium and fluorescence images were acquired using a confocal fluorescence microscope (Nikon Eclipse Ti A1-A; Nikon Instruments Inc.). All acquisition parameters (laser intensity, gain, exposure time, and pinhole settings) were kept constant across experimental conditions.

### 2.10. Quantification of Nrf2 Nuclear Translocation

Nrf2 nuclear translocation was quantified using CellProfiler (version 4.2.8, Broad Institute). Nuclei were identified from DAPI-stained images using the IdentifyPrimaryObjects module with Otsu global thresholding. To estimate the cytoplasmic compartment, nuclear masks were expanded by 5 pixels using the ExpandOrShrinkObjects module, and a perinuclear cytoplasmic ring was generated using the IdentifyTertiaryObjects module by subtracting the original nuclear mask from the expanded mask. The expansion radius was empirically optimized to generate a narrow perinuclear region while minimizing overlap with adjacent cells in confluent 661W cultures. The same segmentation and expansion parameters were applied to all experimental groups. Nrf2 fluorescence intensity was measured in both nuclear and perinuclear cytoplasmic compartments using the MeasureObjectIntensity module. For each cell, Nrf2 nuclear translocation was expressed as the ratio between the mean nuclear fluorescence intensity and the mean fluorescence intensity measured within the corresponding perinuclear cytoplasmic ring (N/C ratio). Approximately 80–120 cells were analyzed per microscopic field. Five independent fields were analyzed for the CTRL and HG groups, whereas six independent fields were analyzed for the HG + PIGA-1138 group. For each field, the mean N/C ratio was calculated and used as a single experimental value for statistical analysis. All images were processed in a blinded and fully automated manner using identical segmentation and analysis parameters.

### 2.11. Quantification of Acrolein Fluorescence

For each experimental group, three randomly selected microscopic fields were acquired using a 40× objective. All cells within each field were analyzed, resulting in approximately 35–70 cells per group, depending on cell density. Acrolein immunofluorescence was quantified as corrected total cell fluorescence (CTCF) to account for differences in cell size and background fluorescence. Individual cells were manually outlined using bright-field images in ImageJ software. Cell area and integrated fluorescence density were measured for each cell, while the mean background fluorescence was determined from five cell-free regions surrounding each field. CTCF was calculated using the following formula: CTCF = Integrated Density − (Cell Area × Mean Background Fluorescence). All images were processed in a blinded and fully automated manner using identical segmentation and analysis parameters.

### 2.12. Animals

Wild-type C57Bl/6J mice (male or female, 4–6 months of age) were maintained under a 12 h light/12 h dark cycle with free access to food and water. All experimental procedures complied with the ARVO Statement for the Use of Animals in Ophthalmic and Vision Research, the Italian and European regulations for animal experimentation, and the principles of the 3Rs, and were approved by the Italian Ministry of Health and the Ethics Committee of the University of Pisa (No. 719/2022-PR, November 2022). Based on the a priori sample size calculation (see Statistical Analysis), a minimum of 6 animals per experimental group was required. To account for the expected mortality associated with the streptozotocin (STZ) model (approximately 40%), diabetes was induced in a total of 20 mice (10 males and 10 females), while the healthy CTRL group consisted of 6 mice (3 males and 3 females), for a total of 26 animals (13 males and 13 females). Animals were randomly assigned to the experimental groups using a computer-generated randomization sequence. Block randomization was applied to ensure balanced group sizes and an equal male-to-female ratio. Mice were initially allocated to either the healthy CTRL or diabetic cohort before STZ administration. Following confirmation of diabetes, defined as a blood glucose level ≥ 250 mg/dL and determined by measuring blood glucose in tail vein blood samples using a handheld glucometer 48 h after STZ administration, diabetic mice were randomly assigned to the STZ or STZ + PIGA groups, resulting in 10 mice per group (5 males and 5 females). Blood glucose levels were subsequently monitored every 5–7 days throughout the experimental period to confirm the maintenance of hyperglycemia. All surviving STZ-treated mice reached the predefined glycemic threshold and remained hyperglycemic until the end of the study. No animals were excluded because of unsuccessful diabetes induction, and no insulin or rescue treatment was administered during the experimental protocol. Animals not included in the final analyses were exclusively those lost because of STZ-related mortality. To minimize animal use and comply with the principle of reduction, only the minimum number of animals required for each endpoint was included in the final analysis. Visual acuity was assessed longitudinally in 6 mice per group using the Prusky Water Maze, whereas electroretinogram (ERG) and optical coherence tomography (OCT) analyses were performed in independent cohorts of 4 mice per group. Unless otherwise specified, all in vivo sample sizes refer to independent observations obtained from different animals.

### 2.13. Animal Treatment

Experimental diabetes was induced in mice by a single intraperitoneal (i.p.) injection of STZ (Merck KGaA) [25]. STZ was freshly prepared immediately before use by dissolving STZ powder in ice-cold citrate buffer (0.1 M, pH 4.5) to obtain a final concentration of 45 mg/mL, suitable for a single-dose administration of 150 mg/kg in an injection volume of 100 µL for a 30 g mouse. Starting from day 15 after STZ administration, diabetic mice were treated with PIGA-1138 by daily intraperitoneal injection. PIGA-1138 was dissolved in DMSO at 6 mg/mL, diluted 1:1 (*v*/*v*) with PEG200 (Merck KGaA), and administered intraperitoneally at a dose of 10 mg/kg (injection volume: 100 µL per 30 g mouse), following previous evidence on PIGA-1138 efficacy in a mouse model of experimental autoimmune encephalomyelitis [26]. STZ mice not receiving PIGA-1138 were injected daily with the corresponding vehicle (1:1 (*v*/*v*) DMSO/PEG200) according to the same schedule and administration route; healthy CTRLs, which served as physiological non-diabetic controls throughout the study did not receive citrate buffer as citrate buffer is widely regarded as an inert vehicle for STZ and, at the administered volume, is not expected to induce retinal alterations or influence the endpoints investigated. Further, in a previous study using the same STZ-induced mouse model of DR, healthy mice receiving citrate buffer displayed no changes in retinal structure or function compared with healthy mice not receiving citrate buffer [27]. The treatment was continued until the end of the experimental protocol.

**Figure 1:**
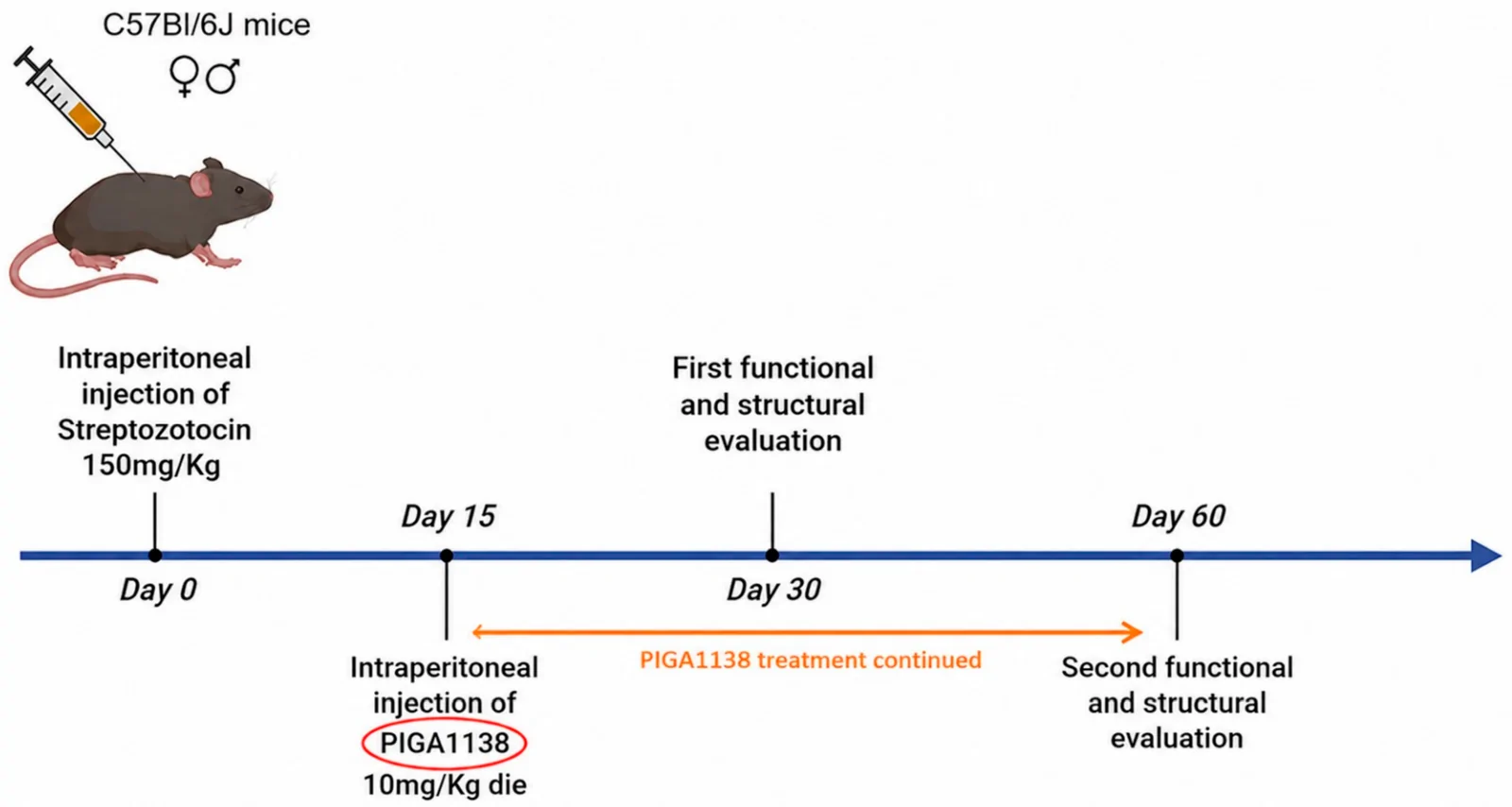
Protocol for the induction of diabetes in C57Bl/6J mice. Mice of both sexes underwent a single injection, given intraperitoneally, of STZ (150 mg/kg). Fifteen days after induction, they were treated daily with intraperitoneal injections of PIGA-1138 (10 mg/kg). Structural and functional evaluations were performed 30 and 60 days after STZ injection.

Functional and structural evaluations were performed on day 30 and day 60 after STZ injection by assessors blinded to group allocations (Figure 1). Body weight and blood glucose levels were monitored throughout the experimental period to confirm the induction and maintenance of diabetes.

### 2.14. Visual Acuity Assessment by Prusky Water Maze

Visual acuity was assessed using the Prusky Water Maze, a two-alternative forced-choice visual discrimination task adapted from Prusky et al. [28]. The behavioural setup comprised a trapezoidal pool equipped with two computer-controlled visual displays positioned at its wider end. The pool was filled with water made opaque by the addition of a nontoxic white tempera to prevent platform visibility and ensure reliance on visual cues alone. The water level was maintained shallow enough for mice to swim without reaching the bottom, and the temperature was kept at approximately 22–25 °C throughout the testing sessions. The behavioral test consisted of three phases: habituation, training, and visual acuity testing.

During the habituation phase, mice were removed from their home cages and placed in close proximity to the rescue platform to facilitate association between the visual stimulus and the platform location. The visual stimulus consisted of vertical sine-wave gratings with a maximum spatial frequency of 0.087 cycles/degree. This phase was repeated three times, with resting intervals between trials. Subsequently, the rescue platform was repositioned on the opposite screen to encourage adaptive responses. After several trials, the release point was progressively moved away from the screen, requiring the animals to swim across the pool to reach the platform.

The training phase was conducted on the following day. Mice were released from the narrow end of the tank and required to swim toward the rescue platform located beneath the screen displaying the visual stimulus. The visual stimulus was alternated between the left and right screens, and the position of the rescue platform was adjusted accordingly to prevent the association of the platform with a fixed spatial location. Mice were considered successfully trained upon achieving at least 80% correct responses in two consecutive trials.

The visual acuity testing phase was performed after completion of the training phase. For each trial, mice were introduced into the narrow end of the tank and used the visual cues displayed on the monitors to locate the hidden escape platform. The spatial frequency of the visual stimulus was progressively increased until the animals were no longer able to reliably discriminate the stimulus. The highest spatial frequency at which the platform could be correctly identified was recorded as the visual acuity threshold. In the event of an incorrect response, the test was repeated until either four consecutive correct responses or seven correct responses out of ten trials were obtained. A maximum of three sessions were conducted per day, each lasting up to 60 min.

### 2.15. Optical Coherence Tomoghaphyand Angiography

Mice were anesthetized with an intraperitoneal injection of Avertin (1.2% Avertin, 0.02 mL/g body weight; Merck KGaA). The avertin was used to provide rapid induction and short-duration anesthesia with a fast recovery, thus minimizing post-anesthesia complications, especially in the case of sensitive diabetic animals [29]. Notably, all the efforts have been made to minimize the established limitations and side effects of this anesthetic regimen. In this respect, avertin solution was freshly obtained following the recommendations of Lieggi and colleagues 2005 [30] to minimize the anesthesia toxicity. In this respect, avertin was prepared by diluting a stock solution (1.6 g/mL tribromoethanol in tert-amyl alcohol) 1:10 in sterile saline to then be stored at 4 °C protected from light and used within 15 days. Moreover, avertin was used to perform Image-guided OICT and ERG analyses with monthly intervals, thus reducing the risk of peritoneal inflammation associated with repeated administration. After anesthesia, mydriasis was induced by 1% tropicamide, and 2% hydroxypropyl-methylcellulose drops were used to avoid eye drying. Thence, mice were laid on a mouse holder allowing the pupil alignment to the optical axis of the probe and bidimensional OCT images were acquired from the right eye by image-guided circular scans (550 µm diameter) around the optic nerve head. The segmentation of retinal layers and the quantification of layer thickness were performed using Insight software version 2.1.7237 (Phoenix Research Laboratories). The segmentation analysis enabled the assessment of the thicknesses of the inner retinal layer (IRL), inner nuclear layer (INL), and outer retinal layer (ORL). The IRL comprised the retinal nerve fiber layer (RNFL), ganglion cell layer (GCL), and inner plexiform layer (IPL), whereas the ORL comprised the outer plexiform layer (OPL), outer nuclear layer (ONL), and photoreceptor outer segments (OS). The IRL and ORL were defined by grouping anatomically related retinal layers to improve segmentation robustness and reduce ambiguity arising from the low contrast and indistinct boundaries between adjacent retinal layers in OCT images. To perform the qualitative macroscopical analysis of the retinal vasculature, mice received an intraperitoneal injection of 50 mg/kg sodium before being laid on a mouse holder and fluorescence images of the eye fundus were acquired concomitantly with the acquisition of OCT images.

### 2.16. Electroretinogram

Mice were subjected to a recording routine including the analysis of the mixed pathway with scotopic electroretinogram (scERG), cone pathway with photopic ERG (phERG), and RGC activity with pattern ERG (pERG). After overnight dark adaptation, the mice were anesthetized by an intraperitoneal injection of Avertin (1.2% Avertin, 0.02 mL/g body weight; Merck KGaA). In light of the possible influence of anaesthesia on ERG responses, the use of Avertin was carefully evaluated based on its minimal impact on retinal activity, as also demonstrated by recent systematic analyses, together with its ease of preparation and administration, and its ability to induce short-term anaesthesia with prompt recovery [31]. The influence of the anaesthesia, if any, can be excluded as a source of intergroup variation by delivering a dosage of avertin proportional to body weight.

After being anaesthetized, mice were gently restrained in a custom-made stereotaxic apparatus allowing an unobstructed visual field and constant body temperature (37 °C). Loop-shaped silver/silver chloride recording electrodes were carefully laid on the corneal surface using micro manipulators. A stainless-steel needle was instead used as a reference electrode, subcutaneously inserted on the mouse cheek. An additional stainless-steel needle was inserted at the tail root and used as a ground electrode. All the ERG responses were recorded and analyzed using a commercially available ERG setup (Retimax Advanced, CSO, Firenze, Italy). ERG routine for each mouse provided the sequential recording of scERG, phERG and pERG from the right eye of each mouse. scERG was elicited by delivering three consecutive flash stimuli at 10 cd·s/m^2^ by means of a Ganzfeld bowl. Signals recorded were averaged to reduce noise after 5000-fold amplification and 1–100 Hz band-pass filtering. The average scERG waveforms were analyzed by measuring the amplitude of the a-wave (baseline to trough), the amplitude of the b-wave (trough to peak), and the implicit time (time to positive peak). Immediately after the scERG session, the mice were adapted to 40.0 cd·s/m^2^ rod-saturating light for 1 min. Then, cone-driven phERG responses were recorded by delivering 50 consecutive flashlight stimuli at 5 cd·s/m^2^ over the same adaptation background. The phERG responses were amplified (5000-fold) and band-pass filtered (1–100 Hz). The average phERG responses were calculated by measuring the b-wave amplitude (baseline to peak) and the photopic negative response (PhNR) amplitude (baseline to trough). Finally, pERG recordings were acquired by delivering contrast-reversing black and white bar stimuli (98% contrast; 1 Hz temporal frequency) by means of a 19″ light-emitting diode display (area: 74° × 62°) aligned to the mouse cornea and spaced 25 cm from the mouse eye (0.05 cyc/deg spatial frequency). pERG signals were amplified (10,000-fold) and band-pass filtered (1–30 Hz). Overall, the responses deriving from 600 consecutive pattern reversals were averaged to reduce noise contamination. The average pERG responses were calculated by measuring the amplitude from the positive peak to the following negative peak.

### 2.17. Statistical Analysis

Statistical analyses were performed using GraphPad Prism software (version 9.0; GraphPad Software, San Diego, CA, USA). For in vitro experiments, n represents independent biological replicates, whereas for in vivo analyses, n represents individual mice.

Group differences were analyzed using one-way ANOVA when assessing the effect of treatment, and two-way ANOVA when evaluating the effects of treatment, time, and their interaction. For the Prusky water maze test, visual acuity was measured in the same mice at 30 and 60 days after STZ injection, constituting a repeated-measures design; therefore, data were analyzed using two-way repeated-measures ANOVA. In contrast, ERG and OCT assessments did not involve longitudinal follow-up of the same animals. Consequently, data from the two time points were treated as independent cohorts and analyzed using two-way ANOVA for non-repeated measures. Before performing statistical analyses, homogeneity of variance was assessed using the Brown–Forsythe test, while normality of the data and/or residuals was assessed using the Shapiro–Wilk test. When variances were not homogeneous, ANOVA was still considered appropriate in the presence of balanced sample sizes and normally distributed data and/or residuals. Pairwise group comparisons were performed using Tukey’s multiple-comparisons test following one-way ANOVA to maintain appropriate control of type I error, or Bonferroni’s post hoc test following two-way ANOVA to provide a stringent control of the family-wise error rate given a limited number of pre-specified comparisons across experimental groups and time points.

In line with the 3R principles, in vivo procedures were designed to use the minimum number of mice per group while ensuring adequate statistical power for reliable interpretation of the observations. In this respect, *a priori* calculation of the sample size was performed using G Power 3.1.9.7 in order to obtain an α = 0.05 and a statistical power ≈0.8 with a large and biologically meaningful effect size of f = 0.7, based on previous observations on the murine STZ model [32]. Therefore, the sample size calculation indicated the use of at least *n* = 6 mice for the repeated measurement design (Prusky water maze) and *n* = 4 mice for time-independent observations (OCT and ERG). Thence, we considered an *n* = 6 per experimental group that was further increased to *n* = 10 for the diabetic groups, to account for a conservative mortality rate of 40%, with a 1:1 male/female ratio. Although we experienced an overall dropout of 30% (2 males, 1 female) in the STZ group and 10% in the STZ + PIGA group (1 male), the number of animals involved in the in vivo analyses remained unaltered, as the dropout remained within the provided mortality rate.

Data are presented as mean ± SEM. Differences were considered statistically significant when *p* < 0.05. When appropriate, data were normalized to the CTRL group and expressed as a percentage of CTRL values.

## 3. Results

### 3.1. PIGA-1138 Decreases Mitochondrial Dysfunction and Apoptosis In Vitro After High Glucose Exposure

**Figure 2:**
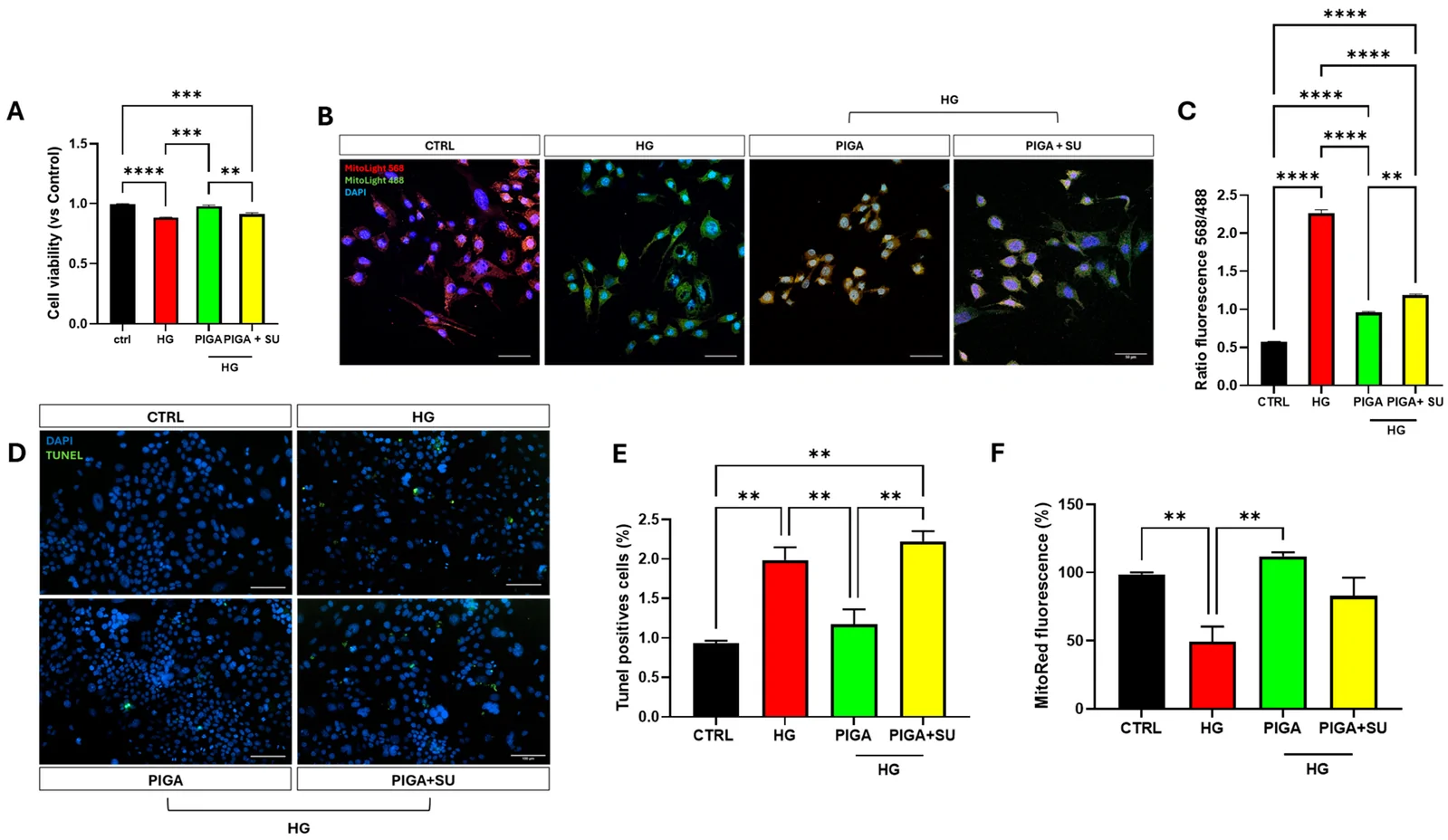
**The protective effect of PIGA-1138 (PIGA) depends on mitochondrial preservation.** CTRL: untreated cells; HG: high glucose (55 mM); PIGA: HG + PIGA-1138 (3 µM); PIGA + SU: HG + PIGA-1138 (3 µM) + SU10603 (3 µM). (**A**) Relative cell viability (*n* = 3 independent experiments); (**B**) Representative images of 661W cells stained with MitoLight. Scale bar: 50 μm. (**C**) Quantification of MitoLight ratio 488/568 of 661W cells (60–180 cells); (*n* = 3 independent experiments). (**D**) Representative TUNEL staining of 661W cells under different conditions. Apoptotic cells appeared in green, nuclei in blue (DAPI). Scale bar: 100 μm. (**E**) Quantification of TUNEL-positive cells (% with respect to the total number of cells). (**F**) MitoTracker Red fluorescence analysis (*n* = 3 independent experiments). Data were expressed as mean ± SEM. (**A**) ** *p* = 0.0023 (PIGA vs. PIGA + SU); *** *p* = 0.0004 (CTRL vs. PIGA + SU); *** *p* = 0.0002 (HG vs. PIGA); **** *p* = 0.0001 (CTRL vs. HG). (**C**) ** *p* = 0.0032 (PIGA vs. PIGA + STZ); **** *p* = 0.0001 (HG vs. PIGA + SU); **** *p* = 0.0001 (HG vs. PIGA); **** *p* = 0.0001 (CTRL vs. PIGA + SU); **** *p* = 0.0001 (CTRL vs. PIGA); **** *p* = 0.0001 (CTRL vs. HG). (**E**) ** *p* = 0.0097 (CTRL vs. HG); ** *p* = 0.0020 (CTRL vs. PIGA + SU); ** *p* = 0.0074 (HG vs. PIGA); ** *p* = 0.0010 (PIGA vs. PIGA + SU). (**F**) ** *p* = 0.0049 (CTRL vs. HG); ** *p* = 0.0010 (HG vs. PIGA). One-way ANOVA followed by Tukey’s post hoc test.

To investigate whether TSPO modulation by PIGA-1138 protects 661W photoreceptor-like cells from HG-induced damage, cell viability, apoptosis, and mitochondrial function were assessed. Cell viability analysis (Figure 2A) showed that HG exposure significantly reduced cell viability compared to CTRL. Treatment with PIGA-1138 significantly improved cell viability compared with HG-treated cells, bringing viability values close to those observed in CTRL conditions. To investigate whether the protective effects of PIGA-1138 depended on TSPO-mediated neurosteroidogenesis, cells were co-treated with PIGA-1138 and the pregnenolone metabolism inhibitor SU10603, an inhibitor of 17α-hydroxylase/C17–20 lyase, also known as P450c17 or CYP17A1 [33], which prevents the conversion of pregnenolone into dehydroepiandrosterone (DHEA); as shown in Figure S1, treatment of 661W cells with SU10603 alone did not reduce cell viability, confirming the absence of any intrinsic cytotoxic effect of the inhibitor. The co-treatment with PIGA-1138 and SU10603 reduced the protective effect observed with PIGA-1138 alone, suggesting that the activity of PIGA-1138 may be, at least in part, dependent on signaling mediated by pregnenolone-derived steroids.

Since mitochondrial dysfunction plays a central role in oxidative stress–mediated damage, ΔΨm was evaluated using MitoLight staining. Representative images and quantification are shown in Figure 2B,C. HG exposure significantly caused a shift from red (healthy mitochondria) to green (impaired mitochondria/apoptotic cells), indicative of mitochondrial membrane damage; treatment with PIGA-1138 significantly preserved ΔΨm, while cotreatment with SU10603 partially reversed the activity of PIGA-1138.

To further investigate whether mitochondrial preservation translated into reduced apoptotic cell death, TUNEL staining was performed. Representative fluorescence images are shown in Figure 2D, while the TUNEL+ cell count is shown in the bar graph in Figure 2E. HG exposure significantly increased the number of TUNEL-positive cells compared with CTRL, indicating increased apoptotic cell death under HG conditions. Notably, treatment with PIGA-1138 significantly reduced TUNEL-positive cells compared to HG-treated cells, supporting its anti-apoptotic effect. Cotreatment with SU10603 abolished the activity of PIGA-1138.

To further confirm the involvement of mitochondrial function and its link to pregnenolone synthesis, ΔΨm was also assessed by quantitative MitoRed fluorescence analysis, measured by Ensight (Figure 2F). HG exposure significantly decreased mitochondrial fluorescence intensity compared to CTRL, indicating mitochondrial membrane depolarization. Treatment with PIGA-1138 restored mitochondrial signal, confirming its protective effect on mitochondrial integrity. Importantly, this effect was abolished in the presence of SU10603.

### 3.2. PIGA-1138 Exerts Neuroprotective Effects In Vitro by Activating Antioxidant Signaling Pathways

**Figure 3:**
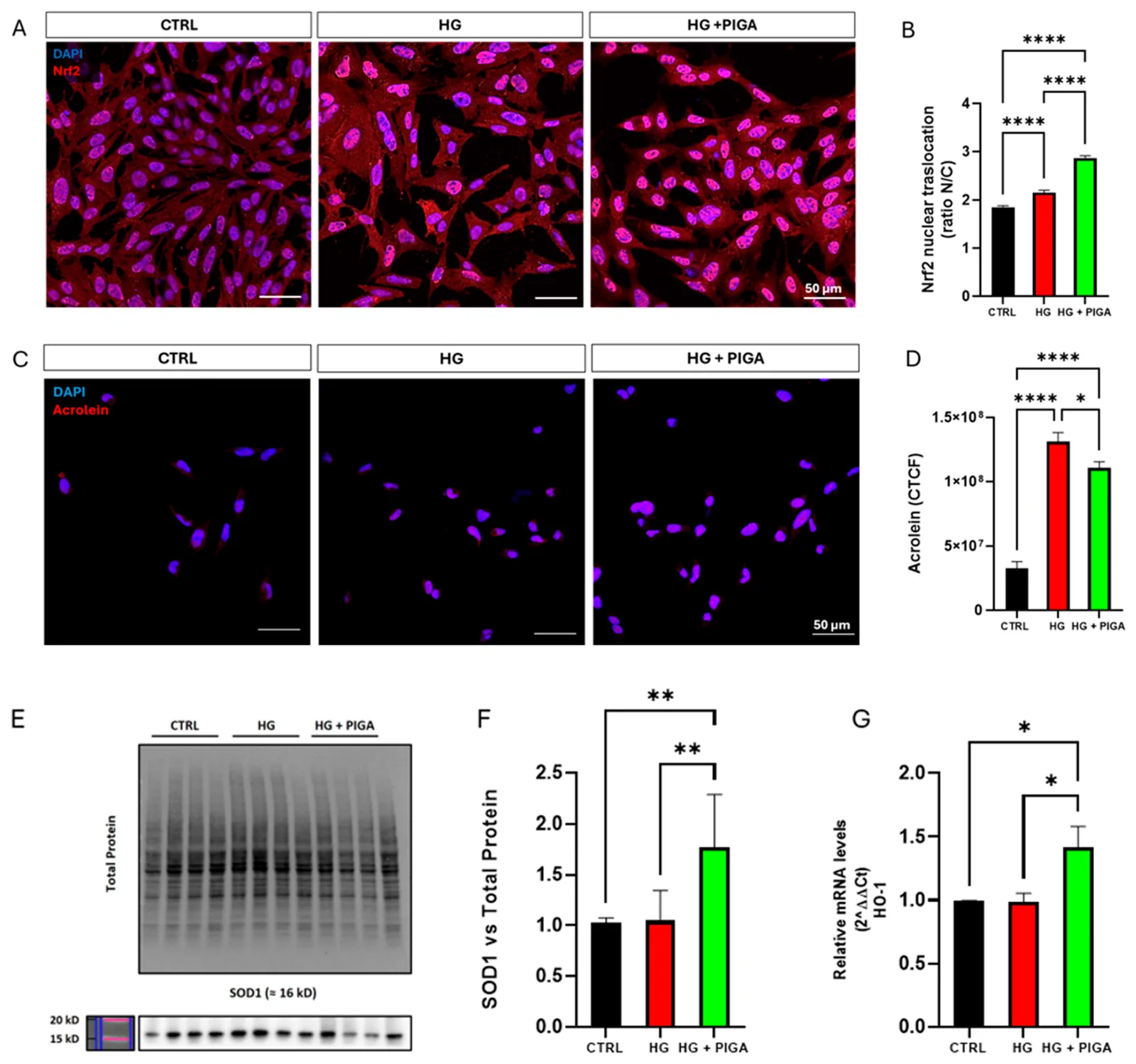
**Effect of PIGA-1138 (PIGA) on antioxidant response and nuclear factor erythroid 2-related factor 2 (Nrf2) activation under HG conditions.** CTRL: untreated cells; HG: high glucose (55 mM); HG + PIGA: HG + PIGA-1138 (3 µM). (**A**) Representative immunofluorescence images showing Nrf2 (red) and nuclei (4,6-diamine-2-phenylindole, DAPI, blue) in CTRL, HG, and HG + PIGA conditions. Scale bar: 50 µm. (**B**) Quantification of Nrf2 nuclear translocation in 661W cells (80–120 cells). (**C**) Representative immunofluorescence images showing Acrolein (red) and nuclei (DAPI, blue) in CTRL, HG, and HG + PIGA conditions. Scale bar: 50 µm. (**D**) Quantification of Acrolein in 661W cells (35–70 cells). (**E**) Representative total protein staining and immunoblot of superoxide dismutase (SOD)1 (~16 kDa) in CTRL, HG, and HG + PIGA conditions (uncropped blot in Figure S2). (**F**) Quantification of SOD1 protein levels normalized to total protein (*n* = 4 independent experiments). (**G**) Relative HO-1 mRNA expression levels (2^−ΔΔCt^) (*n* = 4 independent experiments). Data were expressed as mean ± SEM. (**B**) **** *p* = 0.0001 (CTRL vs. HG; CTRL vs. HG + PIGA; HG vs. HG + PIGA); (**D**) **** *p* = 0.0001 (CTRL vs. HG; CTRL vs. HG + PIGA); * *p* = 0.0335 (HG vs. HG + PIGA); (**F**) ** *p* = 0.0038 (CRTL vs. HG + PIGA); ** *p* = 0.0059 (HG vs. HG + PIGA). (**G**) * *p* = 0.0397 (CTRL vs. HG + PIGA); * *p* = 0.0356 (HG vs. HG + PIGA). One-way ANOVA followed by Tukey’s post hoc test.

To verify whether the observed protective effects of PIGA-1138 on HG-challenged 661W cells were associated with the activation of antioxidant signaling, we evaluated the nuclear translocation of Nrf2, lipid peroxidation, the levels of SOD1 protein, and the gene expression of HO-1 (Figure 3).

We observed statistically significant Nrf2 nuclear translocation in HG-treated 661W cells. Nrf2 immunofluorescence revealed a significant increase in the nuclear localization of Nrf2 in cells treated with PIGA-1138 compared to those exposed to HG, indicating the activation of the Nrf2 signaling pathway (Figure 3A,B). In order to confirm the activation of antioxidant defense induced by PIGA-1138 treatment, we analyzed the levels of the lipid-peroxidation marker Acrolein (Figure 3C,D). PIGA-1138 treatment significantly decreased Acrolein levels in 661W cells after exposure to hyperglycemic conditions.

In addition, subsequent to Nrf2 activation and Acrolein quantification, Western blot analysis demonstrated that protein levels of the Nrf2 downstream effector SOD1 were significantly higher after PIGA-1138 treatment than in HG-exposed cells, suggesting enhanced antioxidant defense (Figure 3E,F).

Consistently, HO-1 mRNA expression was significantly upregulated in the treated group compared to cells exposed to HG (Figure 3G), supporting increased transcriptional activity of Nrf2.

### 3.3. PIGA-1138 Preserves Visual Function in STZ-Induced Diabetic Mice

**Figure 4:**
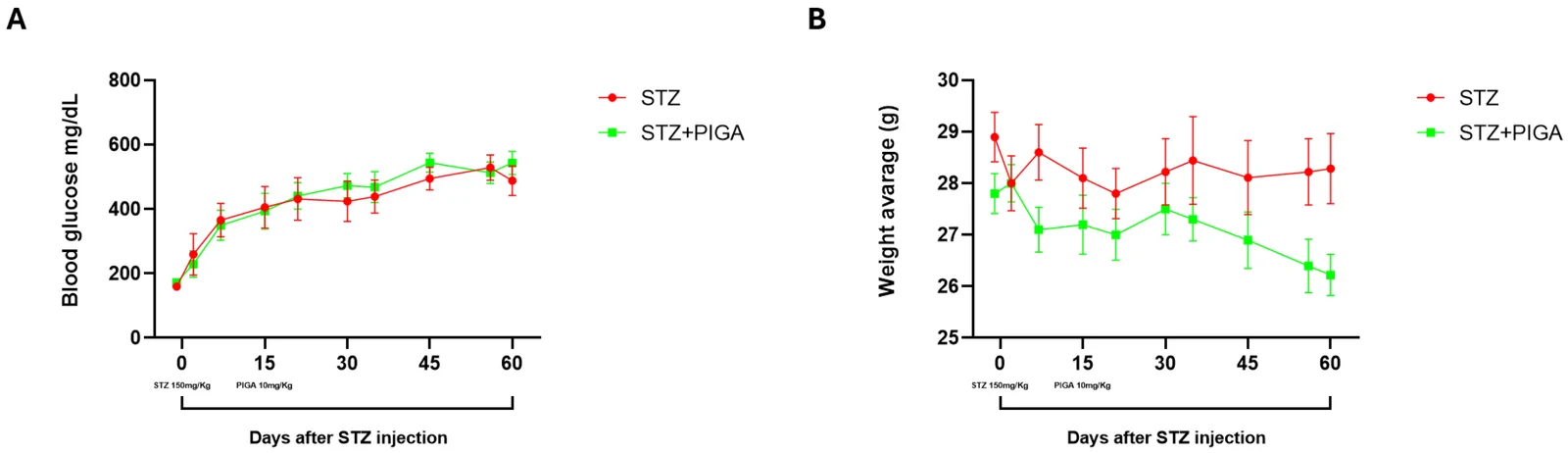
**Longitudinal monitoring of blood glucose levels and body weight in male and female STZ-induced diabetic mice.** (**A**) Blood glucose levels were measured throughout the experimental period in male and female diabetic mice. (**B**) Body weight was monitored over the course of the study in the same animals. Data are presented as mean ± SEM (*n* = 10 animals per group). Statistical analysis was performed using two-way ANOVA followed by Bonferroni post hoc test.

**Figure 5:**
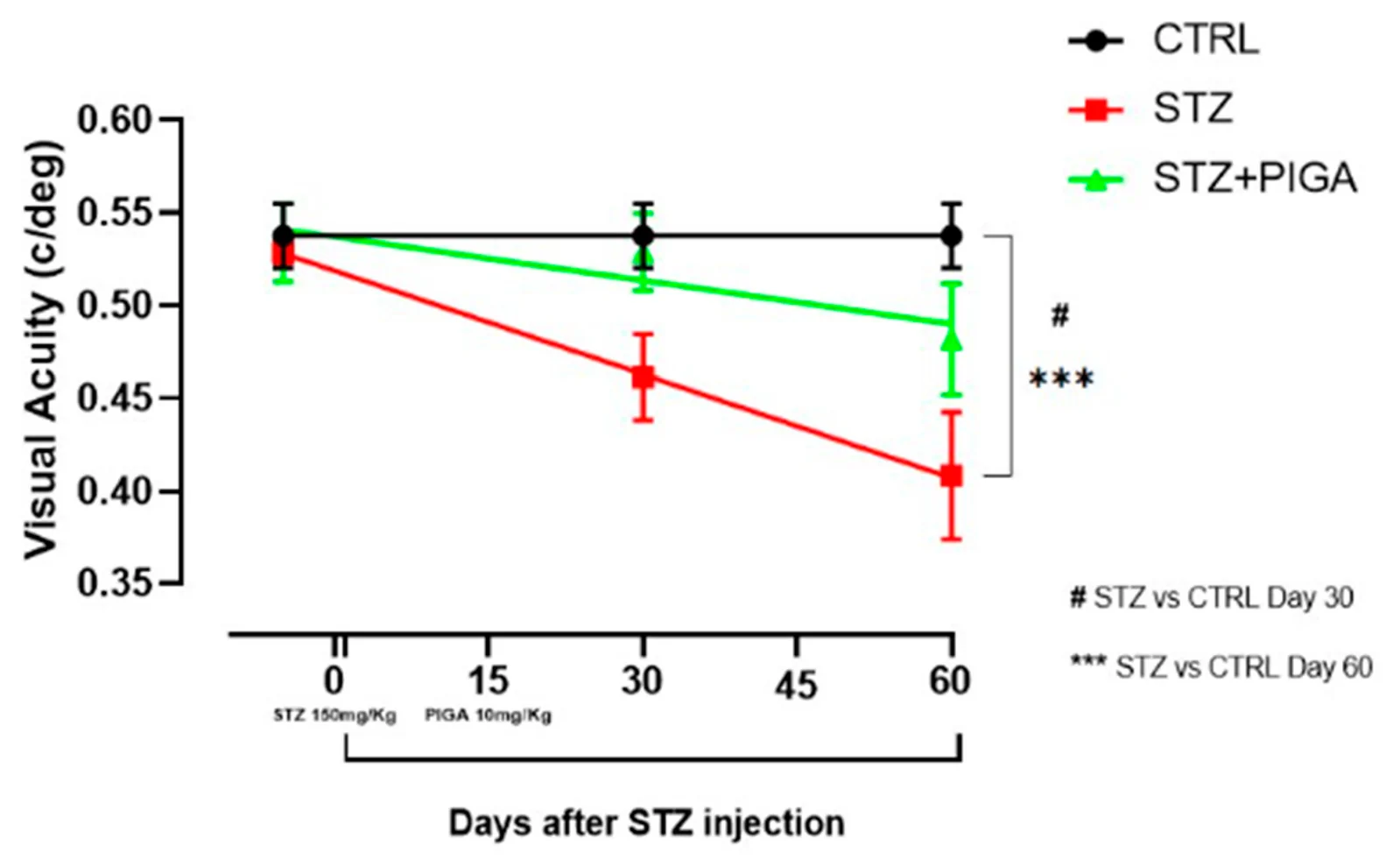
**Longitudinal analysis of visual acuity assessed by the Prusky Water Maze in STZ-induced diabetic mice.** Visual acuity (cycles/degree, c/deg) was measured at baseline (day 0) and at 30 and 60 days in CTRL mice and in diabetic mice untreated or treated with PIGA-1138. Diabetes was induced by streptozotocin (STZ) injection (150 mg/kg), while PIGA-1138 was administered at a dose of 10 mg/kg in the STZ + PIGA group. CTRL mice received no treatment. STZ-treated animals showed a progressive decline in visual acuity over time, whereas PIGA-1138 treatment attenuated diabetes-associated visual impairment. Data are presented as mean ± SEM (*n* = 6 animals per group). *** *p* < 0.001, ^#^ *p* < 0.05 vs. CTRL group. Statistical analysis was performed using two-way ANOVA followed by Bonferroni post hoc test.

To first confirm the successful establishment of the diabetic model, blood glucose levels and body weight were monitored throughout the experimental period (Figure 4). STZ-treated mice developed persistent hyperglycemia that was maintained until the end of the study. PIGA-1138 treatment did not significantly affect blood glucose levels or body weight compared with untreated diabetic mice, indicating that its protective effects were independent of metabolic control. Stratification analysis of blood glucose levels and body weight among diabetic mice allowed us to exclude any sex-dependent significant differences in the overall diabetic phenotype in our experimental setup (Supplementary Figure S3). Visual acuity was longitudinally evaluated using the Prusky Water Maze to assess diabetes-associated visual dysfunction (Figure 5). At baseline (day 0), no significant differences were observed among CTRLs (0.537 ± 0.017 c/deg), diabetic mice (0.529 ± 0.006 c/deg), and diabetic mice receiving PIGA-1138 (0.534 ± 0.021 c/deg), indicating comparable initial visual performance across all experimental conditions.

Following STZ administration, mice showed a progressive decline in visual acuity over time. A significant reduction compared with CTRL animals was already evident on day 30 (CTRL 0.537 ± 0.017 c/deg vs. STZ 0.461 ± 0.023 c/deg, *p* = 0.0466) and further worsened on day 60 (CTRL 0.537 ± 0.017 c/deg vs. STZ 0.408 ± 0.034 c/deg, *p* = 0.0005). Detailed pairwise statistical comparisons among groups at each time point are reported in Table 1. CTRL animals maintained stable visual acuity values throughout the experimental period, confirming the absence of age-related visual impairment under CTRL conditions. In contrast, on day 60, visual acuity in STZ-induced diabetic mice decreased to approximately 0.41 c/deg.

Treatment with PIGA-1138 partially attenuated the STZ-induced decline in visual function. Although differences between diabetic mice and diabetic mice receiving PIGA-1138 do not reach statistical significance (see Table 1), visual acuity values in PIGA-1138-treated mice were consistently higher than those observed in untreated STZ animals across the experimental period. On day 60, mice receiving PIGA-1138 displayed visual acuity values of 0.482 ± 0.030 c/deg compared with 0.408 ± 0.034 c/deg measured in untreated diabetic mice. Notably, visual acuity in the PIGA-1138-treated group was not significantly different from that of healthy CTRL mice at both time points analysed. These results indicate a trend toward preservation of visual function in PIGA-1138-treated mice compared with untreated diabetic animals, suggesting a possible protective effect against diabetes-associated visual impairment.

### 3.4. PIGA-1138 Preserves Retinal Structural Integrity

**Figure 6:**
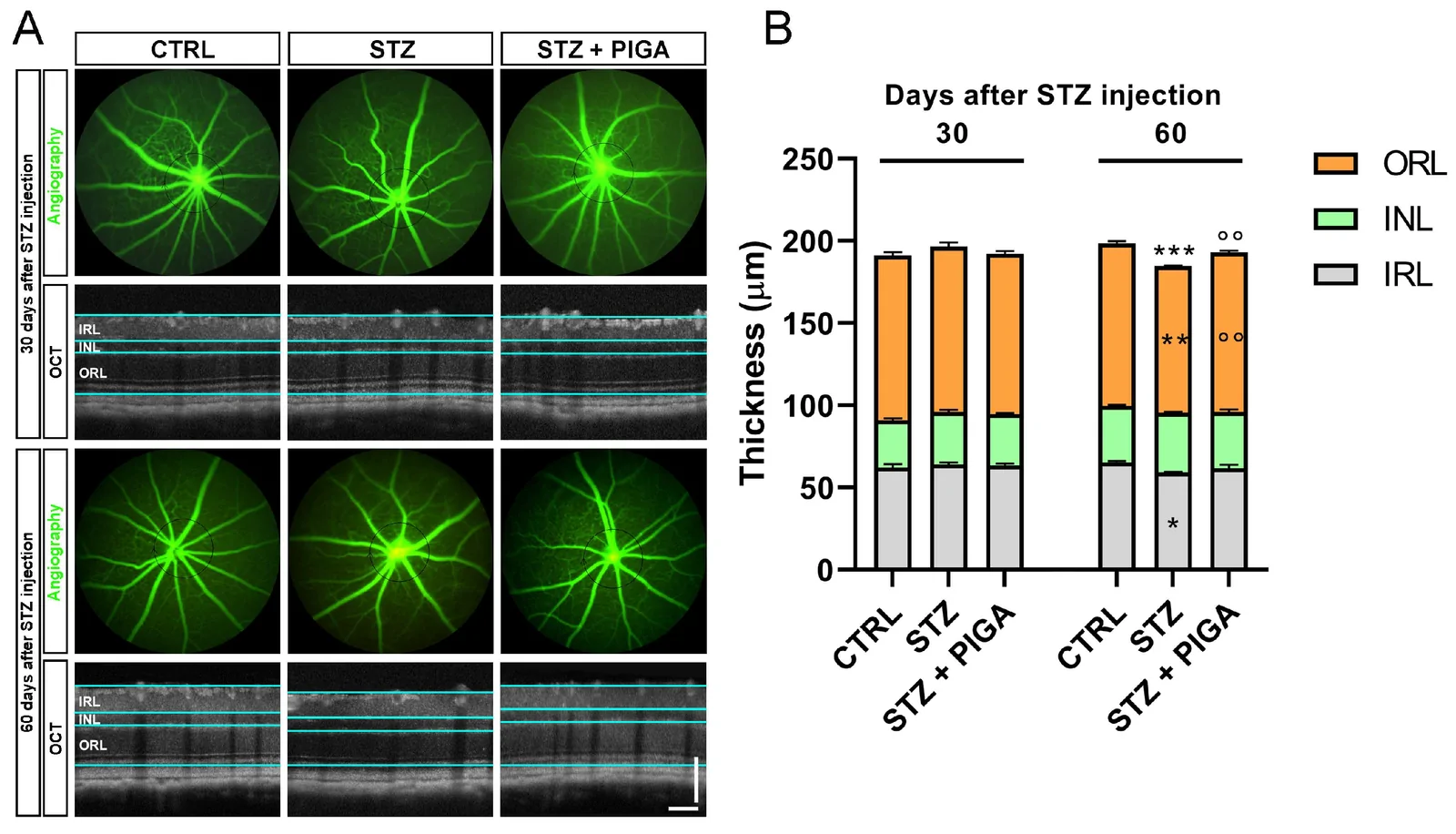
**PIGA-1138 preserves the structural integrity of the retina under diabetes.** CTRL: untreated mice; STZ: mice treated with STZ 150 mg/kg; STZ + PIGA: STZ + PIGA-1138 (10 mg/kg). (**A**) Representative images of fluorescein angiography displaying retinal vessels in the retinal fundus (upper rows) together with OCT images (lower rows) from CTRL mice and STZ-injected mice, either untreated or treated with PIGA-1138. Optical coherence tomography (OCT) images obtained by performing circular b-scans around the optic nerve head were segmented to retrieve the thickness of the inner retinal layer (IRL) (retinal nerve fiber layer + ganglion cell layer + inner plexiform layer), inner nuclear layer (INL), and outer retinal layer (ORL) (outer plexiform layer + outer nuclear layer + photoreceptor outer segment). Scale bar 100 µm. (**B**) Quantification of whole retina, ORL (orange), INL (green), and IRL (gray) thicknesses as obtained from OCT scan images. Data are expressed as mean ± SEM. *n* = 4. * *p* < 0.05, ** *p* < 0.01 and *** *p* < 0.001 versus CTRL; °° *p* < 0.01 versus STZ. Statistical analysis was performed using two-way ANOVA followed by Bonferroni post hoc test.

Retinal structural alterations were assessed by fluorescein angiography and OCT at 30 and 60 days after STZ injection (Figure 6A). Fundus fluorescein angiography did not reveal overt alteration of the vascular network of either untreated or PIGA-1138-treated mice as compared to that of CTRLs. In this respect, no evidence of macroscopic vascular leakage or abnormal vessel outgrowth could be detected, excluding a severe vascular phenotype typical of the advanced stages of DR (Figure 6A, upper rows). Still, as only large hemorrhage or severe changes in capillary perfusion could be detected with sufficient resolution using this technique, the evidence of no overt vascular changes cannot exclude the presence of microscopical vascular alterations such as altered capillary density and tortuosity, pericyte loss and microvascular leakage.

Although there were no overt changes in the retinal vasculature, the OCT analysis revealed significant alterations of the retinal thickness due to diabetes (Figure 6A, lower rows). In this respect, despite no differences in retinal thickness being observed among groups 30 days after diabetes induction, STZ mice at 60 days post-injection exhibited a slight but significant reduction in the whole retina thickness (184.68 ± 0.67 µm, *p* < 0.001 vs. CTRL) compared to CTRLs (198.45 ± 1.69 µm). The retinal thinning observed in the STZ group was associated with a significant reduction of ORL (89.23 ± 0.46 µm, *p* = 0.0017 vs. CTRL) and IRL (59.18 ± 0.53 µm, *p* = 0.024 vs. CTRL) thickness as compared to that of CTRL groups (ORL: 98.79 ± 1.35 µm µm; IRL: 65.35 ± 0.87 µm). The INL thickness in the STZ group (36.26 ± 0.75 µm, *p* = 0.7593 vs. CTRL) was instead comparable to that of the CTRL group (34.33 ± 0.62 µm). The PIGA-1138-treated mice showed a preserved retinal architecture, with a whole retinal thickness (192.96 ± 2.52 µm, *p* = 0.0098 vs. STZ) comparable to that of CTRL animals (*p* = 0.111 vs. CTRL, Figure 6B). Such beneficial effect could be mainly attributed to a significant preservation of the ORL thickness (97.02 ± 1.13 µm, *p* = 0.0095 vs. STZ). No significant preservation of IRL thickness could instead be observed following PIGA-1138 administration as compared to the STZ group (61.79 ± 2.01 µm, *p* = 0.6658 vs. STZ), although a slight tendency could be observed from the loss of statistical difference as compared to CTRLs (*p* = 0.3048).

### 3.5. PIGA-1138 Preserves Scotopic and Photopic ERG Responses

**Figure 7:**
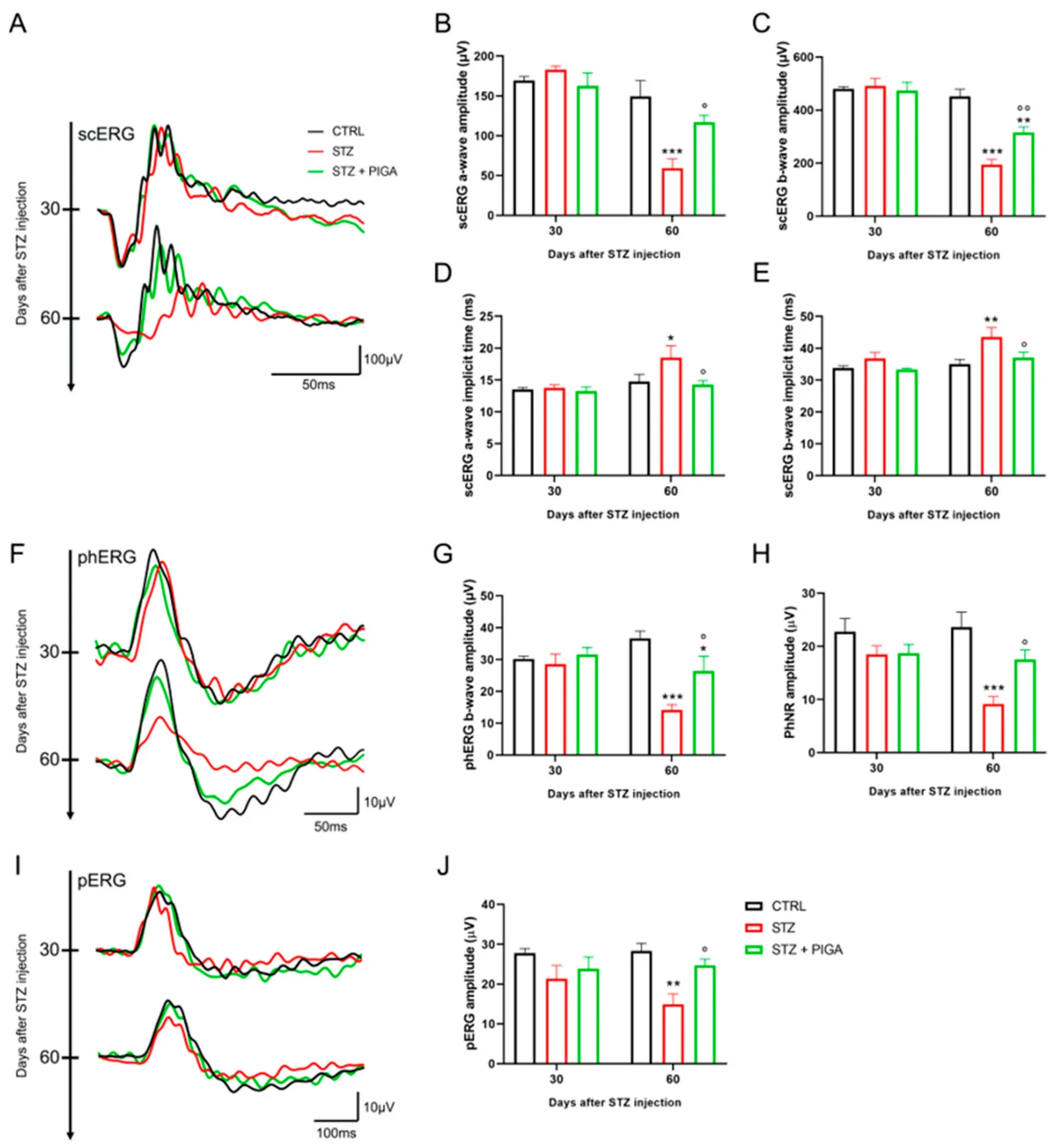
**Longitudinal analysis of DR-related functional alterations following the administration of PIGA-1138.** CTRL: untreated mice; STZ: mice treated with STZ 150 mg/kg; STZ + PIGA: STZ + PIGA-1138 (10 mg/kg). (**A**) Representative scotopic electroretinogram (scERG) waveform recorded in CTRL mice and in STZ-injected mice either untreated or treated with PIGA-1138. (**B**,**C**) Quantitative analysis of (**B**) scERG a-wave and (**C**) scERG b-wave amplitudes reflecting dark-adapted photoreceptoral and post-receptoral activity, respectively. Quantitative analysis of scERG a-wave (**D**) and scERG b-wave (**E**) implicit times. (**F**) Representative photopic ERG (phERG) waveforms and relative quantification of (**G**) phERG b-wave and (**H**) photopic negative response (PhNR) reflecting cone-related post-receptoral activity and retinal ganglion cell (RGC)-related activity. (**I**) Representative pattern ERG (pERG) waveforms and (**J**) quantitative analysis of pERG amplitude related to the selective activity of RGCs. Data are expressed as mean ± SEM. *n* = 4. * *p* < 0.05, ** *p* < 0.01 and *** *p* < 0.001 versus CTRL; ° *p* < 0.05 and °° *p* < 0.01 versus STZ. Statistical analysis was performed using two-way ANOVA followed by Bonferroni post hoc test.

As shown in Figure 7, retinal function was assessed among experimental groups using an ERG routine providing the recording of scERG, phERG and pERG. As shown in Figure 6A, no overt differences in scERG waveforms could be detected among groups 30 days after STZ injection, while an evident decrease in scERG responses of untreated STZ mice could be noticed at 60 days post-injection. The quantitative analysis of scERG parameters, at 60 days post-injection, revealed a significant decrease in the amplitude of both scERG a-wave (59.14 ± 11.92 µV, *p* = 0.0002 vs. CTRL), related to the photoreceptoral activity, and scERG b-wave (193.95 ± 19.79 µV, *p* = 0.0001 vs. CTRL), related to the post-receptoral activity of the retina, in STZ-induced diabetic mice as compared to CTRLs (scERG a-wave: 149.22 ± 20.08 µV; scERG b-wave: 451.62 ± 26.90 µV). The treatment with PIGA-1138 significantly preserved both a- and b-wave amplitudes as compared to untreated diabetic mice (scERG a-wave: 116.69 ± 8.94 µV, *p* = 0.014 vs. STZ; scERG b-wave: 315.25 ± 20.99 µV, *p* = 0.006) despite the b-wave resulted still significantly lower than in CTRL animals (*p* = 0.002; Figure 7B,C). The preservation of scERG amplitudes following PIGA-1138 administration correlated with the amelioration of scERG implicit times of a- and b-wave (Figure 7D,E). In this respect, the delay of both a-wave (18.50 ± 1.85 ms, *p* = 0.043 vs. CTRL) and b-wave peak (43.50 ± 2.96 ms, *p* = 0.0084 vs. CTRL) in diabetic untreated mice at 60 days post-injection as compared to CTRLs (a-wave: 14.75 ± 1.11 ms; b-wave: 35.00 ± 1.47 µV), was prevented after the administration of PIGA-1138 (a-wave: 14.25 ± 0.63 ms, *p* = 0.019 vs. STZ; b-wave: 37.00 ± 1.68 ms, *p* = 0.049 vs. STZ).

Accordingly, 60 days after STZ injection, phERG responses were also altered in untreated diabetic mice compared with CTRLs (Figure 7F). In this respect, the quantitative analysis revealed a significant loss in the amplitude of both phERG b-wave (14.11 ± 1.74 µV, *p* < 0.0001 vs. CTRL; Figure 7G), related to the cone-driven post-receptoral response, and PhNR (9.16 ± 1.39 µV, *p* = 0.0003 vs. CTRL Figure 7H), related to RGC activity, as compared to CTRLs (phERG b-wave: 36.63 ± 2.29 µV; phNR: 23.63 ± 2.82 µV). The treatment with PIGA-1138 attenuated diabetes-driven alterations of phERG response, with both phERG b-wave (26.39 ± 4.57 µV, *p* = 0.015 vs. STZ) and PhNR (17.55 ± 1.77 µV, *p* = 0.027 vs. STZ) significantly higher than those in untreated diabetic mice.

The analysis of RGC activity with pERG, as a sensitive measure of the output activity of the retina towards the brain, further confirmed the trend observed for scERG and phERG (Figure 7I). In this respect, while no alterations in pERG responses could be detected 30 days after STZ injection, an overt loss of pERG amplitude was evident in untreated diabetic mice at 60 days after STZ injection (14.92 ± 2.63 µV, *p* = 0.0029 vs. CTRL; Figure 7I) as compared with CTRLs (28.28 ± 1.96 µV). Administration of PIGA-1138 to diabetic mice completely prevented the diabetes-induced reduction in pERG responses, with pERG amplitude comparable to that of CTRL animals (24.65 ± 1.62 µV, *p* = 0.030 vs. STZ). Overall, these findings demonstrate that PIGA-1138 exerts a protective effect on both rod- and cone-mediated retinal responses under hyperglycemic conditions, preserving retinal output to the brain. Moreover, these data are consistent with the behavioral results obtained from the Prusky water maze test, indicating that preservation of retinal neurons is associated with an improvement in visual function.

## 4. Discussion

The present study demonstrates that PIGA-1138, a high-affinity TSPO ligand, exerts significant neuroprotective and antioxidant effects in both in vitro and in vivo models of DR. Our findings suggest that targeting TSPO represents a viable strategy to mitigate the neurodegenerative component of DR, which is increasingly recognized as a primary pathological event alongside microvascular dysfunction [13].

TSPO was first identified in peripheral tissues as a receptor for benzodiazepine [34]. The expression of this cholesterol translocator was then detected in many organs, including the retina, in which the expression is weak in healthy conditions and increases in pathological states; activating TSPO has been found to exert neuroprotective effects [35]. In particular, an increase in TSPO expression has been found in the retina of STZ-induced diabetic rats [36,37] and in peripheral blood mononuclear cells of DR patients [38,39]; however, no data are available on possible effects of TSPO modulation in this retinal disease. PIGA-1138 is a chemical belonging to the class of *N*,*N*-dialkyl-2-arylindol-3-ylglyoxylamides that has been proven to induce neurosteroidogenesis in rat and human astrocytic models [21]. It has been demonstrated that the interaction of PIGA-1138 with TSPO reduces oxidative stress and prevents neurodegeneration in 661W cells challenged with lipopolysaccharide and in a mouse model of retinitis pigmentosa [14,22].

Hyperglycemia-induced mitochondrial dysfunction is a central driver of retinal neuronal loss. Here, we used 661W photoreceptor-like cells to evaluate the effects of PIGA-1138 on HG-induced cell damage. The 661W is an immortalized cell line, originally isolated from mouse retinal tumors [40], which is widely used to study the effects of hyperglycemia on retinal cells challenged with HG. Indeed, although photoreceptor loss is not a main feature observed in DR patients, 661W cells are often used as an in vitro proxy for studying the molecular mechanisms of retinal diseases, including DR [41]. Our in vitro data on 661W cells showed that PIGA-1138 significantly improves cell viability and reduces apoptosis induced by HG. Crucially, the protective effect of PIGA-1138 was partially abolished by the pregnenolone metabolism inhibitor SU10603 [14,42]. SU10603 inhibits the enzyme converting pregnenolone into DHEA. The latter plays a major role in steroidogenesis and acts as a neurosteroid in the retina, protecting this tissue from damage. In fact, low serum levels of DHEA have been associated with DR in patients suffering from type 2 diabetes, suggesting that low levels of DHEA may contribute to DR pathogenesis [43]. In addition, DHEA dose-dependently prevents glucose-induced toxicity in bovine retinal pericytes [44]. Moreover, the intravitreal injection of DHEA is able to protect the retina from AMPA-induced excitotoxicity in the rat [45]. Among several mechanisms of action, which include the interaction with many membrane receptors, the beneficial effects of DHEA also rely on its antioxidant activity [46]. Taking all this into consideration, our results suggest that stimulation of mitochondrial neurosteroidogenesis contributes, at least in part, to the protective effects of PIGA-1138 on cell survival, maintenance of mitochondrial integrity, and reduction in oxidative stress. Furthermore, PIGA-1138 preserved the ΔΨm, preventing depolarization typically induced by chronic HG. Indeed, retinal cells’ exposure to HG, as in the case of DR, induces mitochondrial membrane depolarization following the excessive stimulation of the electron transport chain, thus resulting in excessive ROS production and in alterations of mitochondrial structure and function [47]. In this scenario, PIGA-1138 likely preserves mitochondrial activity and ability to produce neurosteroids by maintaining stable ΔΨm.

A key highlight of this study is the characterization of the antioxidant signaling pathways triggered by TSPO modulation. Our data showed that PIGA-1138 treatment significantly enhanced the nuclear translocation of Nrf2, activating the transcription of its downstream target genes aimed at enhancing the enzymatic antioxidant defense, as demonstrated by the upregulation of downstream antioxidant effectors, including HO-1 and SOD1. From a mechanistic point of view, TSPO modulation has been recently demonstrated to trigger Nrf2 activation by reducing the levels of the Nrf2 negative regulator Keap1 in cardiomyocytes, through a mechanism that involves the autophagic flux regulatory protein p62 [48]. In the retinal context, and in line with our present results, TSPO modulation leads to Nrf2 activation and increased levels of Nrf2 target gene expression in human retinal pigment epithelial cells and in human retinal microvascular endothelial cells [15,49]. The present findings suggest that the efficacy of PIGA-1138 may derive from its ability to promote Nrf2 nuclear translocation and modulate its transcriptional activity, thereby boosting antioxidant defense pathways that enhance the ability of retinal cells to counteract hyperglycemia-induced cellular damage and preserve mitochondrial integrity. [50].

The therapeutic potential of PIGA-1138 was further validated in the STZ-induced diabetic mouse model. Untreated diabetic mice exhibited a progressive decline in visual acuity, as assessed by the Prusky Water Maze, whereas PIGA-1138-treated mice showed a trend toward preservation of visual function. Importantly, although no statistically significant differences were observed between untreated diabetic mice and PIGA-1138-treated diabetic mice, the latter consistently exhibited visual acuity values that remained closer to those of healthy CTRL animals throughout the study. This trend is consistent with a protective effect of PIGA-1138 on visual function and sustains the potential of the treatment in attenuating diabetes-induced visual impairment. This functional preservation was paralleled by structural findings from OCT analysis, showing that PIGA-1138 significantly attenuated diabetes-induced retinal thinning, thus suggesting that TSPO ligands may interfere with the pathogenic cascade linking oxidative stress to neurodegeneration in DR. These findings are further supported by electrophysiological evidence. ERG recordings revealed a generalized impairment of retinal function in diabetic mice, involving both outer and inner retinal compartments. Specifically, the reduction of scERG a- and b-wave amplitudes and the delays of their related latencies indicate dysfunction of photoreceptors and post-receptoral neurons. In addition, decreased phERG b-wave and PhNR amplitudes reflect alterations in cone-mediated pathways and RGC activity. Consistently, pERG recordings showed a significant reduction in RGC output at 60 days after STZ injection. PIGA-1138 treatment significantly attenuated these functional deficits, preserving both scotopic and photopic ERG responses as well as RGC activity. Notably, the complete prevention of pERG amplitude reduction indicates a strong protective effect on retinal output pathways. Altogether, the electrophysiological data provide a functional correlate to the behavioral results. Since visual acuity relies on the integrity of photoreceptors, inner retinal neurons, and RGC-mediated signal transmission, the preservation of ERG responses is consistent with the trend toward preserved visual function observed in PIGA-1138 treated mice.

Overall, the convergence of structural and electrophysiological data, together with the trend toward preserved visual acuity, supports the neuroprotective effect of PIGA-1138 in DR, highlighting its ability to preserve retinal function under hyperglycemic conditions. Despite the promising findings, several limitations of the present study should be acknowledged. First, although our data support a protective effect of PIGA-1138 in experimental DR, the precise molecular mechanisms underlying its activity require further investigation. In particular, the direct effects of PIGA-1138 on mitochondrial function and bioenergetics remain to be fully elucidated. Second, the in vitro mechanistic studies were performed using 661W photoreceptor-like cells, which represent a valuable model for investigating neuronal responses to hyperglycemic stress, but do not fully recapitulate the complex cellular interactions that characterize DR. Moreover, photoreceptor degeneration is not considered a primary pathological hallmark of human DR, and therefore the translational relevance of these findings should be interpreted with appropriate caution. Third, while OCT analyses demonstrated structural preservation following treatment, the specific retinal cell populations primarily involved in such beneficial effect remain to be identified and will require cell type-specific investigations. Furthermore, because DR is characterized by both neurodegenerative and microvascular alterations, future studies should determine whether PIGA-1138 also exerts protective effects on the retinal vasculature and other diabetes-associated microvascular complications. Finally, although both male and female mice were included in accordance with current recommendations to improve translational relevance, the present study was not statistically powered to evaluate sex-dependent differences in treatment response. Dedicated studies specifically designed to address potential sex-specific effects of PIGA-1138 will therefore be necessary. Addressing these aspects will provide a more comprehensive understanding of the therapeutic potential of PIGA-1138 and support its further development as a candidate treatment for DR.

## 5. Conclusions

The present study provides evidence that the TSPO ligand PIGA-1138 attenuates key pathological features of experimental DR, likely through mechanisms involving the preservation of mitochondrial functions, the enhancement of Nrf2-mediated antioxidant defenses, and the maintenance of neurosteroid-dependent cell survival. These effects were associated with the preservation of retinal layer thickness and visual functions in diabetic mice. While the precise molecular mechanisms, cell type-specific actions, and potential effects on microvascular pathology remain to be fully elucidated, our findings support TSPO as a promising therapeutic target for DR and provide a strong rationale for further investigating PIGA-1138 as a potential disease-modifying strategy. Future studies addressing these aspects, including possible sex-dependent differences in treatment response, will be important to better define its translational potential.

## Figures and Tables

**Table 1:** Pairwise statistical comparisons of visual acuity among experimental groups (^#^ significant for Day 30; *** significant for Day **60).**

Time Point	Comparison	*p*-Value
Day 30	CTRL vs. STZ	^#^ 0.0466
Day 30	STZ vs. STZ + PIGA	0.0927
Day 30	CTRL vs. STZ + PIGA	>0.9999
Day 60	CTRL vs. STZ	*** 0.0005
Day 60	STZ vs. STZ + PIGA	0.0726
Day 60	CTRL vs. STZ + PIGA	0.2480

## Data Availability

Data will be made available on request.

## Supplementary Materials

The following supporting information can be downloaded at: https://www.mdpi.com/article/10.3390/antiox15081000/s1, Figure S1: CTRL: untreated cells; HG: high glucose (55 mM); Sucrose: Sucrose 30 mM; Sucrose + PIGA: Sucrose 30 mM + PIGA-1138 (3 µM); PIGA: PIGA-1138 (3 µM); SU: SU10603 (3 µM). Relative cell viability, including the comparison of the HG group with osmotic control with sucrose. Data are expressed as mean ± SEM (*n* = 3). ** *p* = 0.0031 (HG vs. SU); ** *p* = 0.0044 (HG vs. Sucrose); *** *p* = 0.0002 (HG vs. Sucrose + PIGA); *** *p* = 0.0003 (CTRL vs. HG) *** *p* = 0.0010 (HG vs. PIGA). One-way ANOVA followed by Tukey’s multiple comparisons test. Figure S2: Representative uncropped western blots showing Total protein (A) and Sod-1 (16 kD) (B) protein expression in control (CTRL), Hyperglycemic condition (HG), and Hyperglycemic condition with PIGA1138-treated cells. Figure S3: Sex-stratified analysis of blood glucose levels and body weight average in STZ-induced diabetic mice. (A) Longitudinal blood glucose levels in male and female STZ-treated mice receiving vehicle (STZ). (B) Longitudinal blood glucose levels in male and female PIGA-1138-treated mice (STZ + PIGA). (C) Body weight changes in male and female STZ-treated mice receiving vehicle (STZ). (D) Body weight changes in in male and female PIGA-1138-treated mice (STZ + PIGA). Data are presented as mean ± SEM (*n* = 5 animals per sex and treatment group). No significant sex-dependent differences were observed in either blood glucose levels or body weight throughout the experimental period, indicating comparable induction and maintenance of the diabetic phenotype in male and female mice.

## Abbreviations

The following abbreviations are used in this manuscript:

DR	Diabetic retinopathy
ROS	Reactive oxygen species
TSPO	Translocator Protein
Nrf2	Nuclear factor erythroid 2-related factor 2
SOD1	Superoxide dismutase 1
HO-1	Heme oxygenase 1
HG	High glucose
DMEM HG	High-glucose Dulbecco’s Modified Eagle’s Medium
DMSO	Dimethyl sulfoxide
CTRL	Control
PFA	Paraformaldehyde
PBS	Phosphate-buffered saline
DAPI	4,6-diamidine-2 -phenylindole
ΔΨm	Mitochondrial membrane potential
BSA	Bovine serum albumin
N/C ratio	Ratio between the mean nuclear fluorescence intensity and the mean fluorescence intensity measured within the corresponding perinuclear cytoplasmic ring
CTCF	Corrected total cell fluorescence
ERG	Electroretinogram
OCT	Optical coherence tomography
i.p.	Intrepaeritoneal
STZ	Streptozotocin
IRL	Inner retinal layer
INL	Inner nuclear layer
ORL	Outer retinal layer
RNFL	Retinal nerve fiber layer
GCL	Ganglion cell layer
IPL	Inner plexiform layer
OPL	Outer plexiform layer
ONL	Outer nuclear layer
OS	Photoreceptor outer segments
scERG	Scotopic electroretinogram
phERG	Photopic electroretinogram
pERG	Pattern electroretinogram
PhNR	Photopic negative response
DHEA	Dehydroepiandrosterone
