# Supplementary material for: TSPO Modulation by PIGA-1138 Attenuates Oxidative Stress and Preserves Retinal Function in Experimental Diabetic Retinopathy
Source: Antioxidants (Basel). 2026 Aug 12;15(8):1000. doi: 10.3390/antiox15081000 (PMC13509178; doi:10.3390/antiox15081000)
Supplement: Supplementary file 1 [file antioxidants-15-01000-s001.zip › antioxidants-4333122-supplementary.pdf]

Supplementary Materials

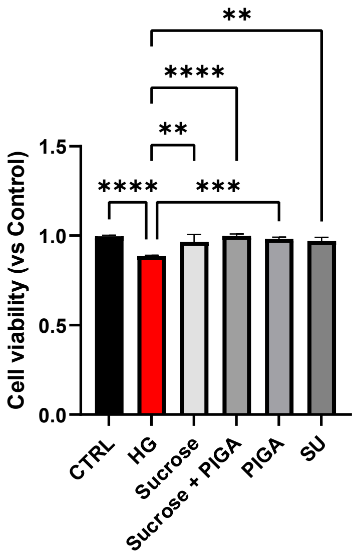

Figure S1. CTRL: untreated cells; HG: high glucose (55 mM); Sucrose: Sucrose 30mM; Sucrose + PIGA: Sucrose 30mM + PIGA-1138 (3 μM); PIGA: PIGA-1138 (3 μM); SU: SU10603 (3 μM). Relative cell viability, including the comparison of the HG group with osmotic control with sucrose. Data are expressed as mean ± SEM (n = 3). **p=0.0031 (HG vs SU); **p=0.0044 (HG vs Sucrose); ***p=0.0002 (HG vs Sucrose + PIGA); ***p=0.0003 (CTRL vs HG) ***p=0.0010 (HG vs PIGA). One-way ANOVA followed by Tukey's multiple comparisons test.

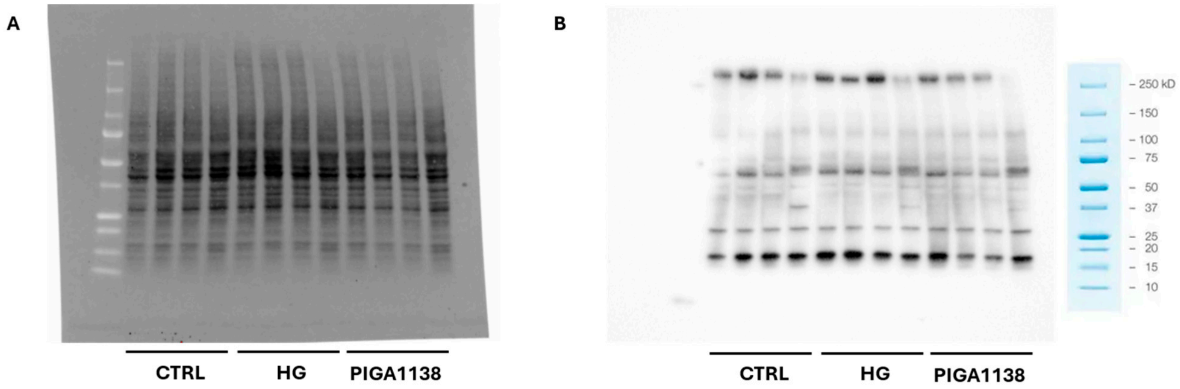

Figure S2. Representative uncropped western blots showing Total protein (A) and Sod-1 (16 kD) (B) protein expression in control (CTRL), Hyperglycemic condition (HG), and Hyperglycemic condition with PIGA1138-treated cells.

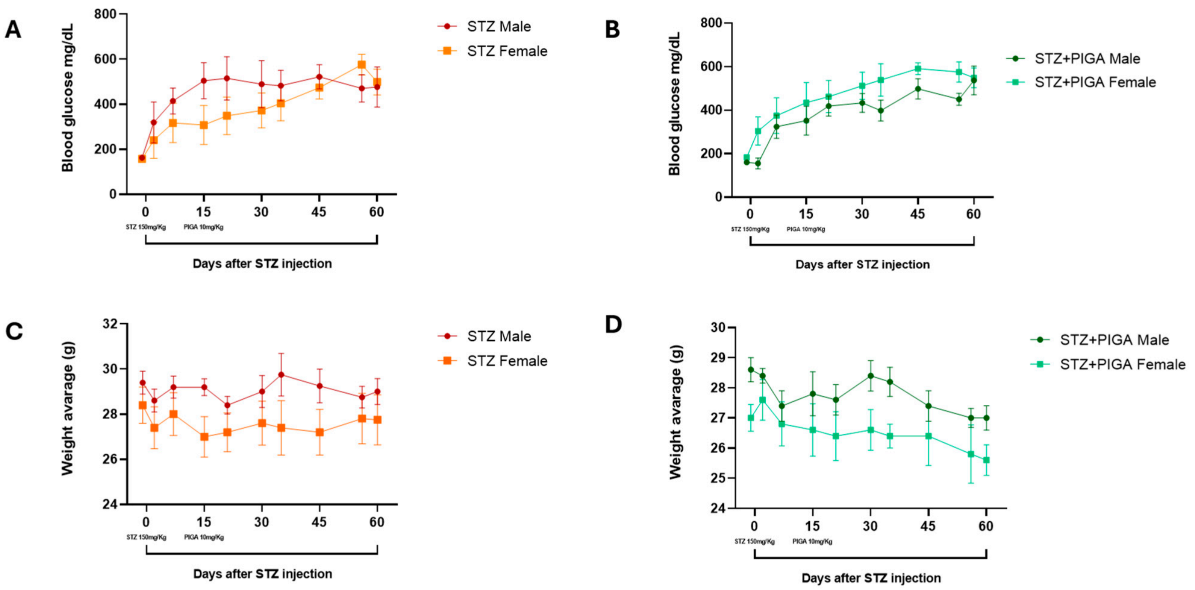

Figure S3. Sex-stratified analysis of blood glucose levels and body weight average in STZ-induced diabetic mice. (A) Longitudinal blood glucose levels in male and female STZ-treated mice receiving vehicle (STZ). (B) Longitudinal blood glucose levels in male and female PIGA-1138-treated mice (STZ+PIGA). (C) Body weight changes in male and female STZ-treated mice receiving vehicle (STZ). (D) Body weight changes in in male and female PIGA-1138-treated mice (STZ+PIGA). Data are presented as mean ± SEM (n = 5 animals per sex and treatment group). No significant sex-dependent differences were observed in either blood glucose levels or body weight throughout the experimental period, indicating comparable induction and maintenance of the diabetic phenotype in male and female mice.
